# The Role of Ultrasound in Accessing the Distal Radial Artery at the Anatomical Snuffbox for Cardiovascular Interventions

**DOI:** 10.3390/life13010025

**Published:** 2022-12-22

**Authors:** Alexandru Achim, Orsolya Ágnes Péter, Kornél Kákonyi, Viktor Sasi, Attila Nemes, Călin Homorodean, Agata Stanek, Dan Mircea Olinic, Zoltán Ruzsa

**Affiliations:** 1 Klinik für Kardiologie, Medizinische Universitätsklinik, Kantonsspital Baselland, 4410 Liestal, Switzerland; 2 Medicala 1 Invasive Cardiology Department, University of Medicine and Pharmacy “Iuliu Hatieganu” Cluj-Napoca, 400125 Cluj-Napoca, Romania; 3 Department of Internal Medicine, Invasive Cardiology Division, University of Szeged, 6720 Szeged, Hungary; 4 Vascular Surgery Department, University of Szeged, 6720 Szeged, Hungary; 5 Department and Clinic of Internal Medicine, Angiology and Physical Medicine, Faculty of Medical Sciences in Zabrze, Medical University of Silesia, Batorego 15 St., 41-902 Bytom, Poland

**Keywords:** vascular ultrasound, radial ultrasound, distal radial access, snuffbox approach, transradial approach

## Abstract

In an effort to refine transcatheter vascular interventions, radial artery access has moved more distally at the anatomical snuffbox. Here, more challenges appear as the artery is smaller, more angulated, and more difficult to palpate. Including ultrasound guidance as a mandatory step during puncture may encourage more operators to switch to this approach. In the femoral approach, ultrasound guidance is strongly recommended because of bleeding complications, whereas in the proximal (conventional) radial approach, the role of ultrasound remains optional, and in current practice, almost all cases are performed by palpation of the pulse only. However, in distal radial access, the situation is different because the artery differs in caliber and position, and imaging can help the operator for a clean puncture, especially since repeated punctures are not only painful but also any hematoma formation leads to the complete compression of the artery and failure of access. The aim of this review is to investigate the rationale of vascular ultrasound during distal radial access and to establish some techniques and anatomical landmarks for the ultrasonographic exploration of the dorsal area of the hand.

## 1. Introduction

Distal radial access (DRA) has recently gained global popularity as an alternative access route for vascular procedures. With this approach, the hand is pronated, and the puncture is performed at the level of anatomical snuffbox where the artery seems to have approximately the same caliber, a superficial approachable trajectory but with a different puncture learning curve. Among the benefits of DRA are the low risk of entry-site bleeding complications and the low rate of radial artery occlusion (RAO) [1]. Antegrade flow to the arm is maintained through the superficial palmar arch branch, and thus the length of the occluded vessel should be limited to the segment between the access site and the origin of the superficial palmar arch [1]. In comparison to the traditional radial approach at the wrist, other possible benefits of distal radial access include improved patient and operator comfort during catheterization and faster hemostasis with a reduction in nursing staff time because this section is more superficial, which lends itself to even faster healing [2]. DRA also plays a special role in chronic total occlusion (CTO) recanalization where dual arterial access is required [3]. Lastly, it preserves the proximal forearm’s radial segment for future interventions or arterial graft harvesting, although the guidelines do not recommend choosing the radial artery as a bypass conduit after catheter manipulation.

The anatomical snuffbox is defined as the triangular depression in the dorsum of the hand, forming a hollow space when the thumb is fully extended. It is bordered by the abductor pollicis longus and extensor pollicis brevis laterally and the extensor pollicis longus medially. The scaphoid and trapezium carpal bones compose the floor. The distal radial artery, cephalic vein, and superficial branches of the radial nerve are all contained within the snuffbox; therefore, caution should be taken when gaining access [1]. This is particularly important when only palpation, rather than ultrasound (US) guidance, is used for cannulation. Radial pulsation is weaker in this region, the learning curve may be longer, and multiple puncture attempts can increase the risk of tendon, periosteum, and radial nerve damage [2]. In current practice, many interventional cardiologists do not routinely use ultrasound, and so the additional requirement of adopting it into use may contribute to the learning curve. Anatomical landmarks can be easily seen by the US, and the operator can choose a nonangulated and nondiseased target segment. Too-distal access should be avoided because wire can travel into the deep palmar arch, where compression is difficult to obtain, or in the princeps pollicis branch, with a risk of thumb necrosis [4]. Anterior single-wall puncture can be performed very safely with this technique, avoiding multiple attempts or double-wall penetration with subsequent hematoma formation. The vessel is very superficial and is usually accompanied by one or two veins of similar size [5]. Compressibility tests can differentiate between the two types, and meticulous scanning can identify the superficial branch of the radial nerve. Ultrasound has an advantage over the tactile location in that the operator can also measure the arterial diameter and determine whether the radial artery can accommodate the required procedural sheath and equipment and choose a smaller diameter sheath in order to reduce the risk of vascular injury, unnecessary pain, and RAO [6]. Therefore, the use of ultrasound during distal access is highly recommended. This is not reflected in the current practice as proximal radial access (PRA) is performed almost completely by the palpation of the pulse, as the arterial trajectory is superficial, and the level of operator comfort and success is high. The role of US in this scenario has no immediate benefit, although some studies have shown some hematoma reduction by guiding the puncture with the help of imaging. As hematoma of the forehand is rarely severe enough to determine compartment syndrome and most of the time the hemostasis is fast and efficient, this additional step was never translated into vascular access guidelines or consensuses. However, this aspect should not immediately mean that the situation with DRA is exactly the same, and the role of US in this novel approach should receive its dedicated analysis. In the interventional radiology community, the use of US is much more widespread and accepted in current practice, and these contemporary practices have begun to be taken over by the cardiology community, especially with the rise of structural interventions that require femoral access (venous or arterial), axillary access, jugular access, etc.

The purpose of this article is to present our single-center learning curve experience with ultrasound-guided DRA, explain some relevant technical considerations, and provide a step-by-step protocol for performing DRA using sonographic and anatomical integration, ensuring safe access and maximizing technical success. 

## 2. Anatomical Considerations

**Figure 1:**
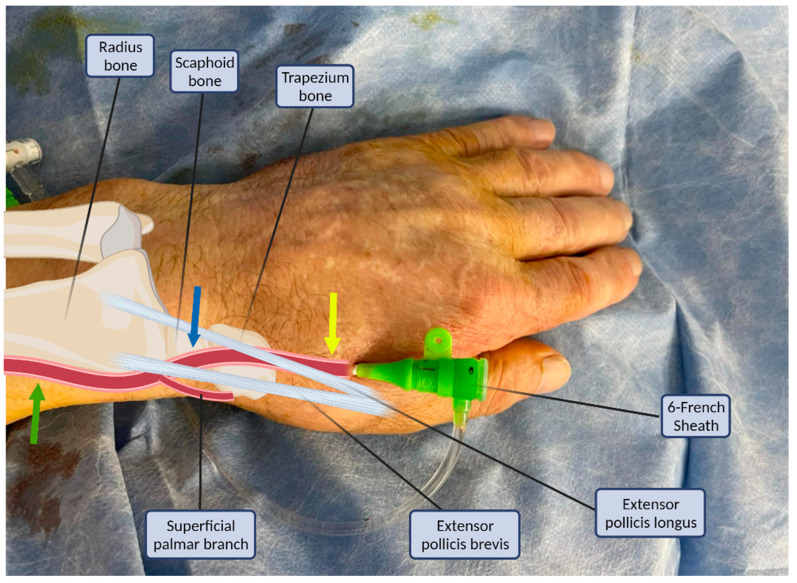
Structures of the anatomical snuffbox in connection with the arterial sheath. Common radial puncture sites: proximal (green arrow), anatomical snuffbox (blue arrow), and distal dorsal (yellow arrow).

The puncture site and the anatomy of the distal radial artery and its surrounding structures are illustrated in Figure 1. The radial artery descends from the lateral side of the forearm above the radius towards the wrist, where it is perceptible between the flexor carpi radialis tendon medially and the anterior border of the radius; this is where the typical transradial access site (PRA) is located [7,8]. The superficial palmar branch of the radial artery arises at the wrist, passing through the thenar muscles and anastomosing with the end of the ulnar artery to produce the superficial palmar arch. Distally, the radial artery continues posterolaterally to pass onto the dorsal part of the wrist. To complete the deep palmar arch, the radial artery anastomoses with the deep branch of the ulnar artery. The two arches are sometimes incomplete or hypoplastic (in approximately 20% of the cases) [9]. 

There are two sites where the pulse of the radial artery in the dorsum of the hand may be palpated and vascular access could be attempted [1]. The first is the anatomical snuffbox which is a triangular depression space on the radial, dorsal aspect of the hand (Figure 1, blue arrow). The second is beyond the extensor pollicis longus, the medial border of the snuff box (Figure 1, yellow arrow) [1]. The anatomical snuffbox is a tridimensional triangle-shaped space with three borders, a floor, and a roof. The floor is a “bone base” composed of the distal radius, scaphoid, trapezium, and the base of the first metacarpal bone. The roof is superficial, composed of skin. The medial and lateral borders are bounded by tendons of the extensor pollicis longus and the extensor pollicis brevis, respectively. The proximal border is formed by the styloid process of the radius. Within this narrow triangular space, various structures are located, including the distal radial artery (RA), a branch of the radial nerve, and the cephalic vein [1,8].

Despite the short accessible length, shallow depth, and limited diameter of the RA in the snuff box with complex surrounding structures, distal radial access through the snuff box has recently been performed [3,4,6,7,10,11]. In the following section, we will discuss how ultrasonography can be practically embedded in the cannulation process.

## 3. Ultrasound Guidance

With the advent of DRA, Kiemeneij et al., who first reported the success rate of this technique in 2017 (89%) [10], published a follow-up review 4 years later advocating for the inclusion of US in the steps of this technique [12]. Choosing US allows the identification of anatomical landmarks and enables accurate vessel access. The authors highly encourage the usage of US, especially at the beginning of the DRA learning curve. The set-up requires no special materials or preparation. In addition to the standard equipment needed for radial artery cannulation, operators need a high-frequency ultrasound machine (e.g., 5 to 10 MHz or higher), a linear array probe (transducer), a sterile water-based lubricant in a single-use packet (preferred over a multi-use bottle of ultrasound gel), a sterile probe cover to seal the probe and the probe cable, and sterile rubber bands (alternatively, the probe may be placed in a sterile glove and the cord wrapped in a sterile drape). 

The probe is placed on the wrist at the level of the anatomical snuffbox. The visualization of the vessels can be improved by adjusting the depth on the ultrasound machine to the minimum depth and adjusting the gain. The examiner’s hand should rest partially on the patient, and only light pressure should be applied to the soft tissues. The vigorous pressing of the transducer leads to the compression of the artery. The artery is visualized as an anechoic or dark circle that can be distinguished from veins based on the pulsation of the vessel. Manual compression, pulsed wave Doppler, and colour flow Doppler are additional tools that ease the detection of the artery. The transverse (short-axis, cross-sectional) ultrasound view is easy to obtain and is best for identifying veins and arteries and their orientation to each other. The long-axis (longitudinal, in-plane) ultrasound view is technically more difficult to obtain, but the entire needle is continuously imaged, ensuring accurate intraluminal placement. At our institution, the longitudinal method is preferred for cannulation. During the early stages of US use, a learning curve is to be expected; however, this should not dissuade operators from using this valuable know-how. Basic expertise in PRA is needed before moving to DRA but no prior US experience is mandatory. One common error is that once the needle punctures the skin, it is no longer helpful to inspect the wrist. The operator should instead watch the ultrasound screen and move the probe to search for the needle tip.

**Figure 2:**
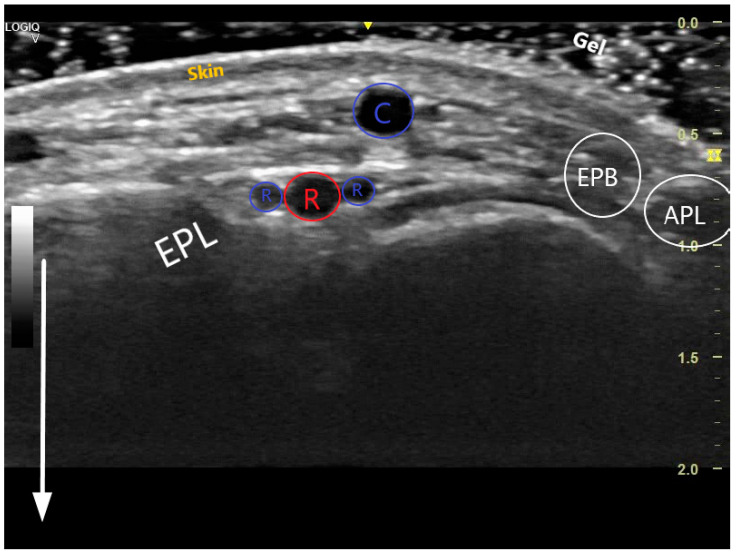
Transversal US image at the anatomical snuffbox. The depth has been adjusted so that the structures near the transducer form the majority of the image. The white arrow demonstrates the near and the far during scanning. The 3 layers of the skin are visible, superficially is the cephalic vein (C), deeper the radial artery (red R) accompanied by 2 radial veins (blue Rs). The snuffbox is surrounded by the extensor pollicis longus (EPL), extensor pollicis brevis (EPB), abductor pollicis longus (APL), and scaphoid (S).

**Figure 3:**
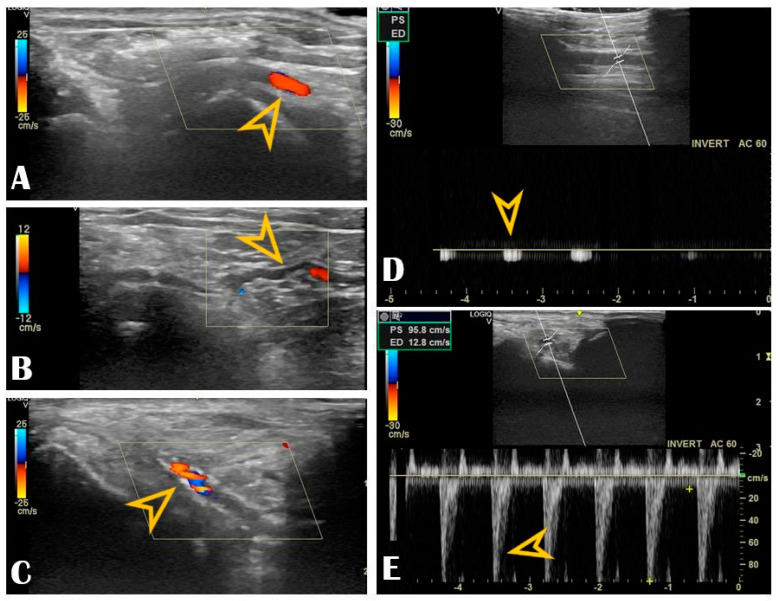
Radial vascular ultrasound findings: Panel (**A**): Normal radial artery (arrow). Panel (**B**): Radial artery tortuosity (arrow). Panel (**C**): Radial artery calcification (arrow). Panel (**D**): Occluded radial artery (the arrow shows the monophasic curve and the decreased peak systolic velocity). Panel (**E**): Radial artery stenosis (the arrow shows the increased peak systolic velocity).

The key anatomical structures that should be identified in the anatomical snuffbox are illustrated in Figure 2. The radial artery should be scanned along the snuffbox in the short-axis view and then switched to the long-axis view in order to select the best site for puncture, aiming for sufficient caliber (ideally > 1.8 mm) and the absence of tortuosity and disease. The common long-axis view is exemplified in Figure 3 along with other pathological variants of reduced or increased peak systolic velocities. The puncture area (the triangle) is separated in two by the tendon of the extensor pollicis longus muscle, which is prominent and easily palpable, and which impels the operator to puncture distal or proximal to it. The true snuffbox area is proximal to the tendon of the extensor pollicis longus muscle, as illustrated in Figure 1 (blue arrow) and Figure 2 where the artery is located laterally to the tendon. The artery then continues under the tendon, actually entering the first intermetacarpal space where it can be palpated and punctured again (dorsum of the hand area—not so preferred among operators, Figure 1, yellow arrow), and finally, it continues its course medially and deeply, forming anastomoses with the ulnar artery. The skin is then punctured parallel to the probe in the exact center of the probe.

## 4. Puncture Technique

**Figure 4:**
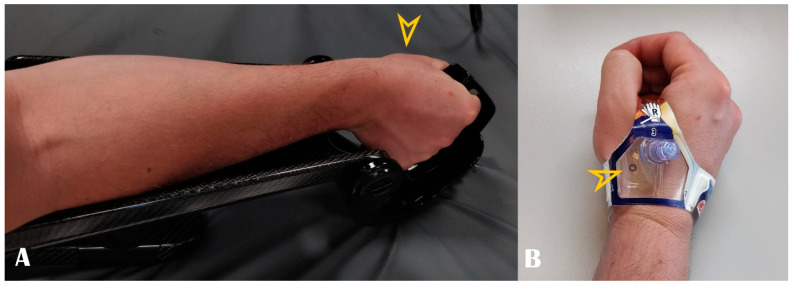
Panel (**A**): Optimal hand positioning during arterial access. Panel (**B**): Example of dedicated compression band. Arrowheads show the puncture site.

A step-by-step description of the puncture technique is shown in Supplementary Materials Video S1 (Online). Briefly, the arm is placed in a pronated position. Slight ulnar deviation and mimicking holding a glass of wine can straighten up the vessel trajectory. Dedicated devices can fix the hand in this position (Figure 4, panel A). This trick is particularly helpful during wire and sheath advancement as the artery travels under the scaphoid bone. In the case of left radial access, the arm is naturally placed over the patient’s abdomen, and the hand is positioned over the right groin. After the subcutaneous injection of 1–3 mL lidocaine 2%, Seldinger’s technique puncture is performed in the anatomical snuffbox. The local anesthetics should be divided into two parts: a small quantity (approx. 1 mL) should be injected directly under the skin, as the artery is superficial and the remaining amount should be kept once the guidewire is in, before inserting the sheath. This division is useful because large amounts of anesthetic can push/compress the artery, and the pulse may be lost. However, this aspect leads us back to one of the advantages of the US: all of these changes are visible and predictable.

As discussed earlier, the distal radial artery may be punctured proximally to the tendon of the extensor pollicis longus muscle in the anatomical snuffbox or distally to it at the dorsum of the hand. An open metal needle for a clean anterior puncture is recommended in order to avoid touching the periosteum. This also avoids spasm and other puncture-related complications such as hematoma or dissection. As the artery is very superficial, immediately under the skin, even a small hematoma can compress the artery and cause further attempts to be more difficult to perform. The vessel can be punctured as long as there is Doppler blood flow visible. In the video, a 21-gauge needle was used for puncturing the desired artery, and 5–6 French sheaths were passed through a 0.018-inch guidewire. No palpation of the artery is required, and the operator watches the US screen. The needle can be inclined more than 45 degrees. In some cases the guidewire may encounter resistance due to tortuosity; leaving the needle free rather than fixing it with the hand may help overcome the acute angles, or even coronary guidewires may be used. A spasmolytic cocktail which consisted of 200–250 micrograms of nitrate and 2.5 mg verapamil was introduced through the sheath together with 2500–5000 IU of unfractionated heparin. Hemostasis was achieved using a combination of one StatSeal Disc (Biolife, LLC, USA) with elastic bandage compression (3–4 h). Alternatively, there are dedicated compression devices as well (Figure 4, panel B), or even a simple compressive bandage is enough. The artery is superficial and therefore easily compressible. Compared to PRA, multiple studies have shown significantly shorter times with DRA to obtain hemostasis [4,11,13].

## 5. Caliber Differences

In general, a difference of 10–20% exists between the proximal and distal segments of the radial artery. However, this difference does not always apply, and there are some variations between studies, populations, ethnicities, sex, constitution, etc. In order to ensure the safety of the procedure and to reduce the risk of RAO, it is recommended that all patients undergo preoperative US to determine the diameter of the distal radial artery and select an appropriate radial artery sheath because the puncture success rate is positively correlated with the diameter of the distal radial artery [14]. Moreover, the size of the radial artery is an important predictor of RAO, although there is still uncertainty about the incidence of RAO with DRA. An attempt was endeavored to find related and predictive factors. Two Korean studies found similar distal radial artery diameters among their population [15,16]. The study of Kim et al. found an average diameter of the distal radial artery of 2.57 ± 0.50 mm, and females had a smaller size (2.40 ± 0.53 mm) as compared to males (2.65 ± 0.46 mm) [15]. Their cohort contained 117 patients [15]. Lee et al.’s population had similar measurements: 2.31 ± 0.43 mm (right distal radial) and 2.35 ± 0.45 mm (left distal radial). The diameters were smaller in women than in men (right: 2.15 ± 0.38 mm vs. 2.43 ± 0.44 mm, *p* < 0.001; left: 2.18 ± 0.39 mm vs. 2.47 ± 0.45 mm, *p* < 0.001) [16]. Female sex, low body mass index (BMI), and low body surface area (BSA) were significant predictors of DRA diameter < 2.3 mm [16]. Among the Indian population, Deora et al.’s study has shown the size of the right distal radial artery to be 2.27 + 0.39 mm in males and 2.09 + 0.38 mm in females. Diabetes, hypertension, height, and weight were important predictors of distal radial diameter [17]. A study from Japan by Norimatsu et al. of 142 patients has shown the diameter of the distal radial artery (2.60 ± 0.34 mm) to be significantly smaller as compared to the proximal radial artery (3.10 ± 0.4 mm), and the difference was seen in both males and females. The same study did not find the diameters to be correlated with age and height, but they were positively correlated with both body weight and BMI [18]. In a Chinese population, Li et al. found an overall mean internal diameter of the distal radial artery of 2.05 ± 0.41 mm and that hypertension and distal radial artery diameter have a positive effect on the success rate of DRA, whereas diabetes and female gender have a negative effect [14]. A study from Canada by Hadjivassiliou et al. in 287 patients undergoing interventional radiology procedures has shown the mean diameter of the proximal radial artery 2.55 ± 0.39 mm and distal radial artery 2.34 ± 0.36 mm (*p* = 0.001), and the difference was significant in both genders [19].

To summarize, the diameter and structure of the distal radial artery can vary significantly, and it is difficult to predict the actual diameter of the artery. Besides sex and small bodies, other comorbidities such as diabetes mellitus can affect the integrity of the radial artery’s wall. There is an overall 2+ mm diameter among all reported populations which safely allows 6-French catheters to be used. With the availability of thinner sheaths by various manufacturers, the same size sheath may be used for DRA by keeping the outer diameter to 6-French and increasing the inner diameter. In order to ensure the safety of the procedure and to reduce the risk of RAO, it is recommended that all patients undergo preoperative ultrasonography to determine the diameter of the distal radial artery and select an appropriate radial artery sheath. Another indirect but not-so-reliable method is to rely on how well and strong the pulse feels when palpated. Finally, the differences between the aforementioned studies could also be explained by the variability in defining the exact place where it is measured (snuffbox versus dorsum of the hand) and the method of measurement (intima to intima, media to media, unintentional compression with the transducer, etc.).

## 6. Benefits, Learning Curve, Different Scenarios

**Figure 5:**
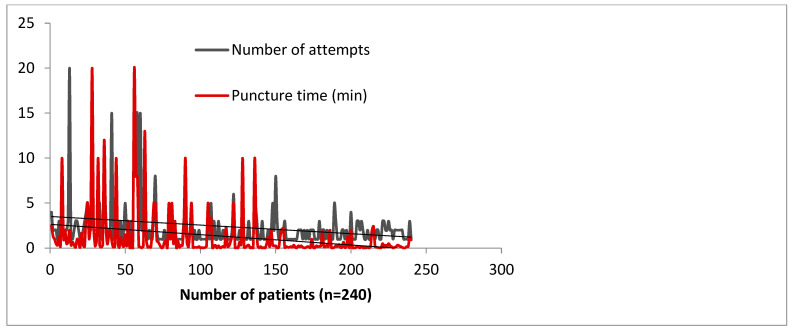
Learning curve impact on puncture time and number of attempts in 240 consecutive subjects, by 4 operators over a period of 3 months—data collected from the Szeged Registry [4].

As with any technique, learning distal radial access is associated with a learning curve. We have analyzed the impact of the learning curve in our high-volume catheterization center, in terms of the number of attempts and the puncture time [4]. Figure 5 shows that a learning curve for distal radial access exists but is an acceptable process [4]. A threshold of 100–150 cases is sufficient for otherwise already skilled operators to establish a reliable procedural method of DRA access. Roh et al.’s team needed at least 100 cases to consistently maintain a high success rate of >96% [20]. These numbers may alleviate the concerns of operators over the feasibility of incorporating DRA access into their standard practice. However, marked improvement can be felt within 50 cases, which is encouraging for a novice operator [21]. Being more distal, the artery is slightly smaller in diameter (0.2–0.3 mm less) but the difference showed no impact on 6–7-French sheaths [22,23].

**Figure 6:**
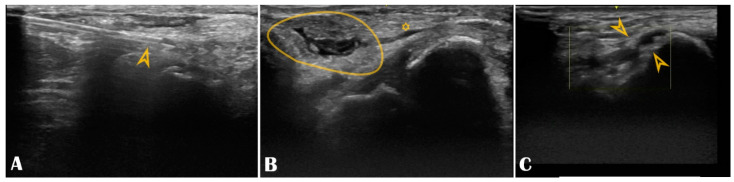
Panel (**A**): The puncture needle is clearly seen in the longitudinal axis (arrowhead); Panel (**B**): Hematoma (circle) compressing the distal radial artery (asterisk); Panel (**C**): The distal radial artery is with stenotic atherosclerotic plaques (arrowheads).

Our center experience reached 1240 consecutive patients who underwent DRA between 2019–2021, all of them under US guidance (from which, 75% of patients received coronary angiography and/or intervention, and the remaining 25% received various peripheral interventions). The extensive results of the registry were published in a multicenter study [4]. DRA was successfully punctured in 97% of all patients, always with ultrasound guidance, with puncture and sheath insertion up to two attempts in the vast majority of patients [4]. We encountered only 2.58% access site crossovers (successful arterial puncture but failed sheath insertion), mainly performed via the ipsilateral conventional PRA, allowing the initial vascular access strategy to be carried out and waiving transfemoral access [4]. Factors leading to crossover were RAO, small vessel diameter, tortuosity, or significant atherosclerosis. The remarkably low crossover rate was due to the pre-procedural US screening that deferred small distal radial arteries. The indication spectrum was broad, from coronary interventions to structural or peripheral. The maximum sheath size inserted was 9F, for transradial balloon aortic valvuloplasty [24]. No major ischemic or bleeding complications were recorded. We have learned along the way that US is useful beyond guiding the puncture. Scanning the artery before the puncture can provide details about the condition of the artery. Findings worthy of mentioning are that multiple attempts lead to needle-induced microhematomas between the intima and media that subsequently narrow or occlude the vessel (by extrinsic compression), giving the operator’s impression that the vessel became “spastic” (Figure 6, panel B). Another peculiar finding observed by our operators during RUS was needle-induced fluid turbulence when penetrating the artery, a highly specific sign of successful puncture (Supplementary Materials Video S1). Plaque formation, calcinosis, reduced lumen, or occlusion are accessible for diagnosis (Figure 6, panel C). A recent study found a significant correlation between radial calcification and coronary calcification; therefore, US may even play a key role in predicting severe coronary artery disease [25]. Moreover, the multivariable logistic regression found a strong correlation between risk factors such as age, smoking, chronic kidney disease, and diabetes mellitus and radial artery calcification [25]. This helps to understand how metabolic systemic diseases affect even the far end of the arterial tree [26,27]. This is of increased importance when the radial artery is potentially considered as a coronary bypass conduit, and the quality of the vessel must be carefully evaluated with US.

In the only study comparing ultrasound-guided DRA versus tactile-guided DRA, ultrasound guidance shifted DRA success from 87% to 97% [28]. The study demonstrated that ultrasound-guided DRA (for diagnostic coronary angiography or PCI) can improve the rate of successful puncture, even though there were no significant differences in procedural outcomes and complication rates [28]. The DISCO RADIAL trial—the largest randomized trial that compared DRA vs. PRA in terms of RAO and that had neutral results— does not mention the rate of US use [29] but positive signals come from another trial, more relevant because it studied the hand function after DRA, demonstrating no impairment, and where the operators used US extensively (85%) [30]. Hemostasis was achieved with inflatable devices in 80% of cases, and the mean hemostasis duration was 123 ± 75 min, which finally led to remarkably low levels of RAO following DRA, further establishing the safety of this access route [30]. Another role of US in assisting DRA was demonstrated in a recent pilot study where chronic RAO was recanalized retrogradely in 30 patients, accessing distally the artery with US, in the only segment where it remained permeable (by collateralization) [31]. Antegrade damped Doppler sign or reverse flow through the palmar arch were positive signs that the puncture could be performed at this level even though the pulse was not palpable [31]. It remains to be seen whether the utility of US in DRA will outweigh its practical role and bring benefits on clinical hard endpoints, as it did for femoral access [32]. For this, it must prove its versatility in acute cases (i.e., ST-elevation myocardial infarction, cardiogenic shock) or in high-risk, complex percutaneous coronary interventions [33,34,35]. Even so, clinical imaging is a safe, cost-effective, easy to learn, and ubiquitously available tool which should be acquired by any operator performing percutaneous interventions—whether they are an angiologist, radiologist, or interventional cardiologist, all need to be “a little” imager as well.

Finally, the role of US in DRA can be extended to postoperative evaluation for RAO (palpation is not a reliable method because the distal radial artery can be collateralized, and its pulse can be palpated even in the presence of upstream occlusion) or for the imaging of DRA vascular complications. US scanning can easily detect pseudoaneurysms and arteriovenous fistulas or check the patency of the artery during follow-up [36]. The majority of trials comparing conventional radial access versus DRA have the RAO rate set as the primary endpoint, which cannot objectively be quantified without US. Additionally, once occluded, the artery can be recanalized using techniques and guides for total coronary occlusions, and here, the evidence of an attenuated flow in the distal segment can be decisive for the operator to succeed in inserting the needle and then the guidewire inside the true lumen of the radial artery [37].

To conclude, it is worth remembering that the transradial approach and devices have improved over the last 28 years, and it is logical to expect that the distal access technique will undergo similar maturation. Almost without a doubt, the method will continue to improve and be condensed even further. The question of whether ultrasound-guided distal radial access should be routinely implemented and performed in everyday practice is still open, but in our view, that is a question well worth pursuing.

## 7. Conclusions

US guidance is a powerful tool for mastering DRA, reducing complications, and maximizing technical success. For more complex percutaneous procedures that require larger catheters (7–8 French), US can also defer small-diameter and/or pathological radial arteries, thus avoiding unnecessary and painful puncture attempts.

## Data Availability

We used PubMed and Web of Science to screen articles for this pictorial review. We did not report any data.

## Supplementary Materials

The following supporting information can be downloaded at: https://www.mdpi.com/article/10.3390/life13010025/s1, Video S1.

## Abbreviations

DRA: distal radial access; RAO, radial artery occlusion; CTO, chronic total occlusion; US, ultrasound; PRA, proximal radial access; RA, radial artery.
